# Effects of Independent and Combined Water-Deficit and High-Nitrogen Treatments on Flag Leaf Proteomes during Wheat Grain Development

**DOI:** 10.3390/ijms21062098

**Published:** 2020-03-19

**Authors:** Dong Zhu, Gengrui Zhu, Zhen Zhang, Zhimin Wang, Xing Yan, Yueming Yan

**Affiliations:** 1 College of Life Science, Capital Normal University, Beijing 100048, China; 18301302142@163.com (D.Z.); zhugary2010@126.com (G.Z.); 2 College of Agronomy and Biotechnology, China Agricultural University, Beijing 100083, China; zhangsirzz@163.com (Z.Z.); zhimin206@263.net (Z.W.); 3 State Key Laboratory of Remote Sensing Science, College of Global Change and Earth System Science, Beijing Normal University, Beijing 100875, China

**Keywords:** wheat, flag leaves, proteome, water deficit, high-N fertilizer

## Abstract

We present the first comprehensive proteome analysis of wheat flag leaves under water-deficit, high-nitrogen (N) fertilization, and combined treatments during grain development in the field. Physiological and agronomic trait analyses showed that leaf relative water content, total chlorophyll content, photosynthetic efficiency, and grain weight and yield were significantly reduced under water-deficit conditions, but dramatically enhanced under high-N fertilization and moderately promoted under the combined treatment. Two-dimensional electrophoresis detected 72 differentially accumulated protein (DAP) spots representing 65 unique proteins, primarily involved in photosynthesis, signal transduction, carbohydrate metabolism, redox homeostasis, stress defense, and energy metabolism. DAPs associated with photosynthesis and protein folding showed significant downregulation and upregulation in response to water-deficit and high-N treatments, respectively. The combined treatment caused a moderate upregulation of DAPs related to photosynthesis and energy and carbohydrate metabolism, suggesting that high-N fertilization can alleviate losses in yield caused by water-deficit conditions by enhancing leaf photosynthesis and grain storage compound synthesis.

## 1. Introduction

Wheat is a major grain crop cultivated worldwide, with over 600 million tons harvested annually. It is a staple food for humans, providing essential amino acids, minerals and vitamins, and beneficial phytochemicals and dietary fiber to human diets [1]. Concerns for food security, meaning the sufficient production and availability of crops like wheat, are rising as the global climate changes and the human population increases [2]. Estimates suggest that food production must increase by 60–110% (from 2005 levels) to adequately feed the global human population by the 2050s [3,4].

Extreme climate events relate to volatility in crop yields, and are thus considered a critical, immediate threat to global crop production. Drought, an extreme weather phenomenon, has significant adverse effects on agricultural production [5,6,7]. Under global climate change and climate warming, the frequency and duration of extreme drought events is increasing faster than previously predicted [8]. Drought stress disrupts cell homeostasis and generally induces a number of morphological, physiological, and biochemical changes in all plant organs [9], particularly affecting leaf photosynthesis and transfer of stored carbohydrates [10]. Under drought stress, plants typically close the stomata, reducing evaporative water loss and decreasing stomatal conductance of carbon dioxide (CO_2_), thereby reducing CO_2_ levels and causing a decline in photosynthetic carbon assimilation and subsequent reduction in crop yield [11,12]. In addition, the remobilization of pre-anthesis stored photoassimilates from photosynthetic organs in wheat is enhanced by water deficit during grain-filling stage, which increases grain-filling rate, accelerates plant senescence and finally results in decline of grain number and weight [10]. According to statistics, a global cereal (maize, rice, and wheat) loss of 1820 million Mg was caused by drought and extreme heat from 1964 to 2007 [5].

Crop growth and development is highly dependent on nitrogen (N), a critical inorganic nutrient for the synthesis of amino acids, chlorophyll, nucleotides, numerous other metabolites, and cellular components. In agricultural production, N availability is the key determinant of crop yield [13], with its critical role in photosynthesis. It has been reported that photosynthesis capability of photosynthetic organs is closely correlated with their N status [14,15,16]. Leaves, the main organs for photosynthesis, thereby create the main organic materials plants need to survive by photoassimilation [17]. During the process of N assimilation, approximate 60–80% of the leaf N is invested in synthesizing the photosynthetic apparatus, particularly Rubisco and light-harvesting complexes, which supports the light-dependent use of CO_2_, water, and inorganic N to produce the basic building blocks of biomass accumulation, such as sugars, organic acid, and amino acid [18,19]. Crop yield is closely related to the net photosynthetic assimilation of CO_2_, where roughly 90% of a plant’s dry weight is derived from photosynthetic carbon assimilation [20]. Therefore, the agricultural use of N fertilizer has been a major achievement in meeting growing food demands by increasing crop production and crop yields in the world, especially in developing countries [21,22]. Statistically, grain production has increased >40% and >55%, respectively, in developed and developing countries [23,24]. 

Northern China, where Chinese wheat production is concentrated, is characterized by arid and semi-arid regimes, where water resources primarily originate from groundwater and rainfall. In this region, annual rainfall ranges from 400 to 800 mm and is concentrated in summer, with insubstantial precipitation during the wheat growing period [25]. This region suffers from drought due to a decreasing water supply and increasing drought frequency caused by climate change [26]. To alleviate losses in crop yields caused by drought conditions, moderately high N fertilizer were generally applied by local farmers in the wheat growing process. In order to achieve high yields and economic benefits under drought conditions, an in-depth understanding of changes in crop growth caused by high N fertilization under water-deficit conditions is required. 

With the rapid development of analytical technology and genomics, proteomics has become a prevalent and powerful tool to characterize systematic changes of crops in response to changing conditions [27]. In the last two decades, numerous drought-responsive proteomic analyses have been conducted in various organs of wheat, including seedling leaf and root [9,28,29,30,31,32,33,34], flag leaf [10], and developing grain [10,35,36,37,38]. Meanwhile, several studies reported the effects of different nitrogen levels on the proteome of wheat seedling leaf [39], root [40], and grain [41,42,43]. However, these studies only focused on the effects of single stressor on wheat proteome; the proteome changes of wheat flag leaf in response to high-N fertilizer under water deficit are still not clear. Here, we conducted the first comprehensive proteome analysis of wheat flag leaves under independent and combined water-deficit and high-N fertilizer conditions. In wheat, flag leaves have the highest photosynthetic efficiency among the photosynthetic organs and serve as the main source of assimilation for grains at later growth stages [44]. Thus, we aimed to describe flag leaf proteome changes and their effects on grain yield under both independent and combined water-deficit and high-N fertilizer conditions.

## 2. Results

### 2.1. Changes in Flag Leaf Physiological Characteristics and Agronomic Traits under Field Treatments

**Figure 1:**
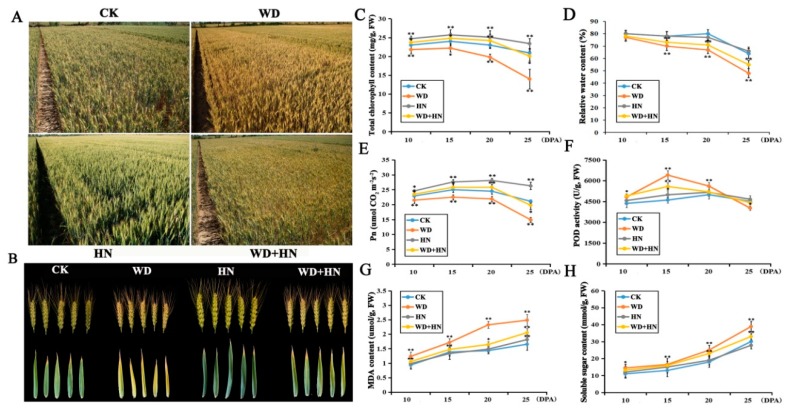
Morphological and physiological parameter changes of bread wheat cultivar Zhongmai 175 under control, water-deficit, high-nitrogen (N) fertilization, and combined treatments. (**A**) The field performance of wheat from different treatment groups at 25 days post-anthesis (DPA). (**B**) The performance of spikes and flag leaves. (**C**–**H**) Physiological parameter changes. CK: control treatment with regular watering and N fertilization; WD: water deficit treatment with no watering and regular N fertilization; HN: high-N treatment with regular watering and high-N fertilization; WD+HN: combined treatment with no watering and high-N fertilization; MDA, malondialdehyde; Pn, net photosynthesis efficiency; POD, peroxidase. Significant differences compared to the control were calculated based on independent Student’s *t*-tests: * p < 0.05; ** p < 0.01.

The water-deficit treatment accelerated plant growth and grain filling, leading to prematurity, whereas the high-N treatment improved vegetative growth and led to unfavorably delayed senescence. Under a water deficit, wheat plants were dwarfed and chlorotic with reduced ears and flag leaves. Under high-N fertilization, plants were robust and green with larger ears and flag leaves. Plants under the combined treatment (water deficit and high-N) displayed an intermediate form between those under independent treatments (Figure 1A,B). 

Physiological parameters of flag leaves showed significant changes under the four treatments. Broadly, total chlorophyll content, relative water content (RWC), net photosynthesis efficiency (Pn), and peroxidase (POD) activity displayed a similar trend of first increasing, then gradually decreasing, during grain development (Figure 1C–F). Relative to the control, the water-deficit treatment resulted in a significant decrease in RWC, chlorophyll content and Pn, whereas the high-N fertilizer and combined treatments showed a significant increase in chlorophyll content and Pn. POD activity was markedly increased in the water-deficit and combined treatments. Malondialdehyde (MDA) and soluble sugar contents showed a gradually increasing trend in all four treatment groups, where both parameters were higher in the water-deficit and combined treatment groups, but no significant difference was found in the high-N treatment relative to the control (Figure 1G,H). In addition, water deficit caused a 4.6% reduce in thousand grain weight (TGW), and ultimately resulted in a 4.6% decrease in grain yield. In contrast, the high-N and combined treatment, respectively, led to 5.2% and 4.1% increase in TGW with ultimate increase in yield of 20% and 6%, relative to the control. 

### 2.2. Flag Leaf Proteome Changes in Response to Water-Deficit, High-N Fertilizer, and Combined Treatments

**Figure 2:**
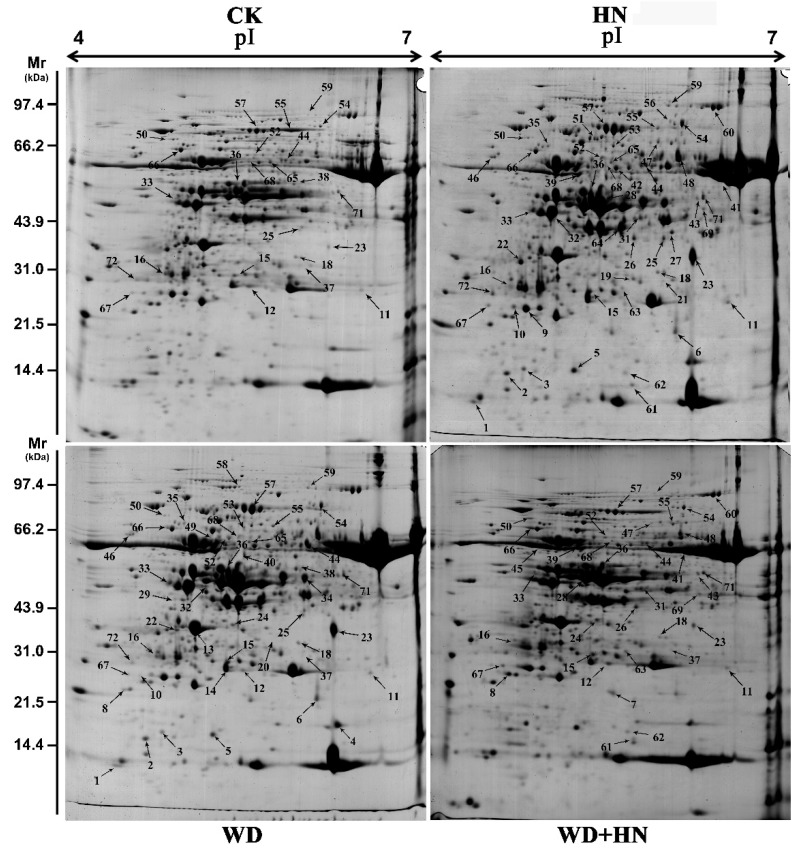
Images from two-dimensional gel analyses of wheat flag leaf proteomes under water-deficit, high-nitrogen (N) fertilization, and combined treatments. Samples were electrofocused on an 18-cm pH 4–7 linear Immobiline DryStrip gel and separated by sodium dodecyl sulfate–polyacrylamide gel electrophoresis on a 12% polyacrylamide gel. All differentially accumulated protein spots are numbered on the gel images. CK: regular watering and N fertilization; WD: no watering and regular N fertilization; HN: regular watering and high-N fertilization; WD+HN: no watering and high-N fertilization.

**Figure 3:**
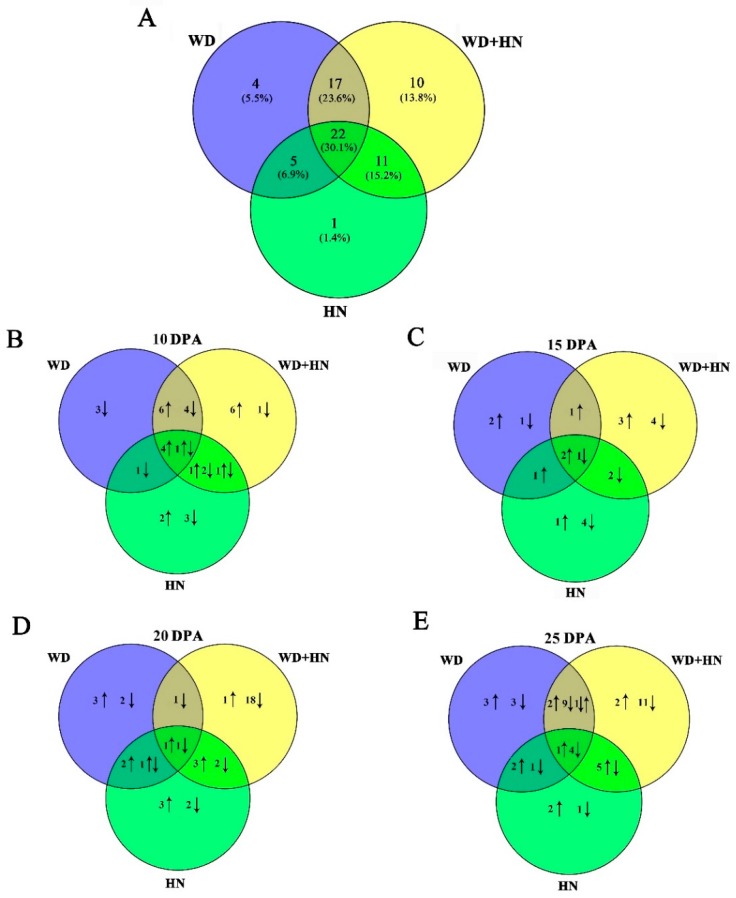
Venn diagrams of 72 differentially accumulated protein (DAP) spots from flag leaves during grain development under water-deficit, high-nitrogen (N) fertilization, and combined treatments. (**A**) Total DAP spots from wheat flag leaves under different treatments. DAP spots at (**B**) 10, (**C**) 15, (**D**) 20, and (**E**) 25 days.

Dynamic proteome changes during four grain development stages under the water-deficit, high-N fertilizer, and combined treatments were detected by two-dimensional gel electrophoresis (2-DE). In total, 72 DAP spots were identified with *p* < 0.05 and expression abundance > 2-fold (Figure 2, Figure S1). Venn diagram analyses of the identified DAP spots indicated that 22 (30.1%) were common in all treatments, while 4 (5.5%), 10 (13.8%), and 1 (1.4%) were specifically expressed in the water-deficit, high-N fertilizer, and combined treatments, respectively (Figure 3A). These results suggest that high N induced the greatest number of uniquely expressed DAP spots among treatments. Observed dynamic changes of DAP spots in flag leaves during all four grain development stages demonstrated that 4 and 1 common DAP spots showed increased and decreased abundance at 10 DPA, respectively (Figure 3B). The number of common DAP spots with increased abundance gradually decreased between 15 and 25 DPA. The number of common DAP spots with decreased abundance were constant at 10 and 15 DPA, but rapidly increased at 25 DPA (Figure 3C–E). The number of DAP spots from each treatment was greatest at 25 DPA, corresponding to significant changes in flag leaf morphological and physiological characteristics at this stage (Figure 1).

### 2.3. Functional Classification and Subcellular Localization of DAPs

All DAP spots were excised from 2-DE gels manually, and then digested with trypsin for further identification by a matrix-assisted laser desorption/ionization mass spectrometer. In total, 72 DAP spots representing 65 unique proteins were successfully identified with a high degree of confidence. Detailed information is provided in Table S1.

**Figure 4:**
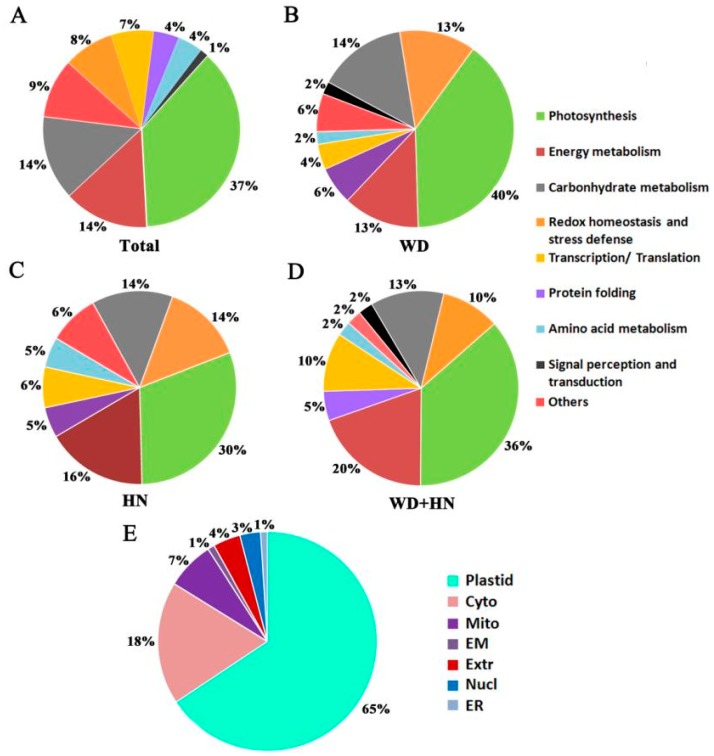
Functional classification and subcellular localization of 72 identified differentially accumulated protein (DAP) spots from wheat flag leaves under water-deficit, high-nitrogen (N) fertilization, and combined treatments. (**A**) Functional classification of total DAP spots. (**B**) Functional classification of DAP spots in the water-deficit treatment (WD). (**C**) Functional classification of DAP spots in the high-N treatment (HN). (**D**) Functional classification of DAP spots in the combined treatments (WD+HN). (**E**) Subcellular localization: Chl, chloroplast; Nucl, nuclear; Cyto, cytoplasm; Extr, extracellular; Mito, mitochondria; EM, endomembrane; ER, endoplasmic reticulum.

Functional classification based on annotation from UniprotKB showed that 62 DAPs were involved in photosynthesis (37%), energy metabolism (14%), carbohydrate metabolism (14%), redox homeostasis and stress defense (8%), transcription/translation (7%), amino acid metabolism (4%), protein folding (4%), signal perception and transduction (1%), and other (9%) (Figure 4A). The three treatment groups showed a similar functional distribution, where DAPs were mainly involved in photosynthesis, and energy and carbohydrate metabolism (Figure 4B–D).

**Figure 5:**
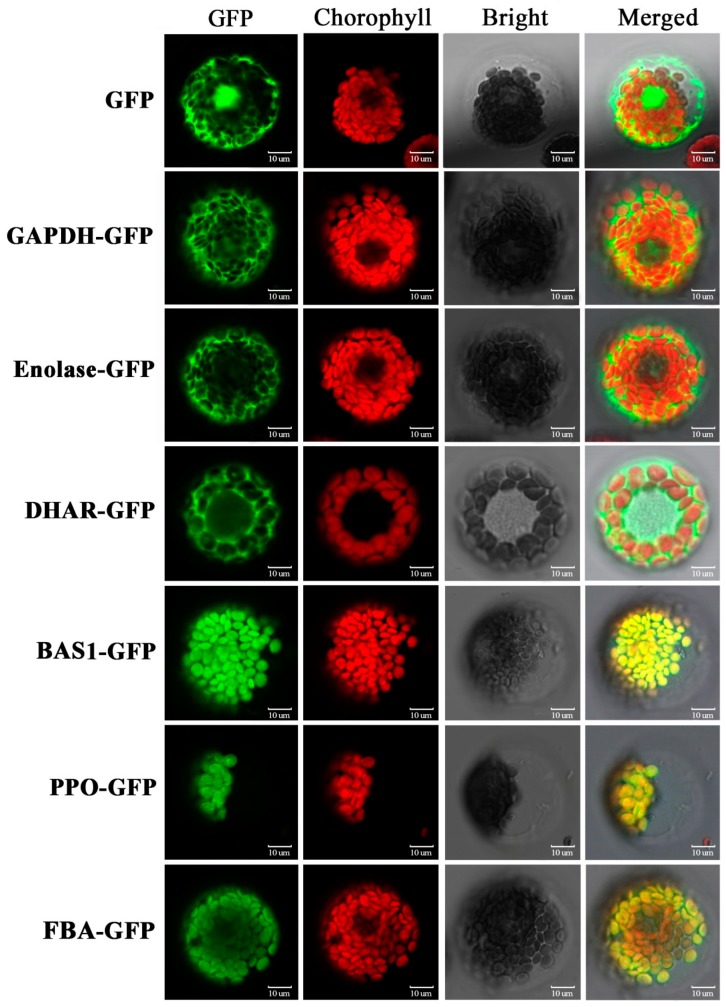
Subcellular localization of selected differentially accumulated proteins (DAPs) by wheat protoplast transformation. GFP: green fluorescent protein signal; Chlorophyll: chlorophyll auto-fluorescence signal; Bright light: bright light field; Merged: combination of GFP, chlorophyll, and bright light. GAPDH, glyceraldehyde 3-phosphate dehydrogenase; DHAR, dehydroascorbate reductase; BAS1, 2-cys peroxiredoxin BAS1; PPO, polyphenol oxidase; FBA, fructose-1,6-bisphosphate aldolase; GFP, negative control.

The subcellular location predication of the DAPs, based on a combination of resources (WoLF PSORT, Plant-mPLoc, CELLO v.2.5, and UniprotKB websites), showed that the majority of DAPs were located in the plastid (65%), followed by cytoplasm (18%), mitochondria (7%), extracellular (4%), nucleus (3%), endomembrane system (1%), and endoplasmic reticulum (1%) (Figure 4E, Table S1). We selected six representative DAPs to verify the website predictions using transiently expressing green fluorescent protein (GFP) fusion proteins in wheat protoplast: glyceraldehyde 3-phosphate dehydrogenase (GAPDH), enolase, dehydroascorbate reductase (DHAR), 2-Cys peroxiredoxin BAS1 (BAS1), polyphenol oxidase (PPO) and fructose-1,6-bisphosphate aldolase (FBA). Strong green fluorescent signals indicated that GAPDH, enolase, and DHAR were located in the cytoplasm, while BAS1, PPO, and FBA were located in the chloroplast. These experimentally derived outcomes were consistent with the website-based predictions (Figure 5, Table S1). 

### 2.4. Dynamic Expression Profiling of DAPs during Grain Development

**Figure 6:**
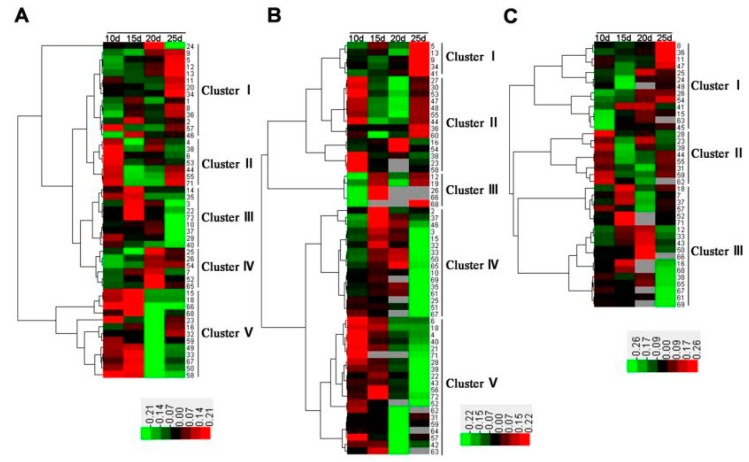
Hierarchical cluster analysis of identified differentially accumulated protein spots in wheat flag leaves from 10, 15, 20, and 25 days post-anthesis under (**A**) water-deficit, (**B**) high-nitrogen fertilizer, and (**C**) combined treatments.

Hierarchical cluster analysis was performed to visualize dynamic expression changes in the identified DAPs. Three hierarchical clusters corresponding to the water-deficit, high-N fertilizer, and combined treatments were constructed using the Euclidean distance method over a complete linkage dissimilarity matrix (Figure 6). In total, 13 expression clusters were classified among 72 DAP spots, including five (clusters I–V) in both the water-deficit (Figure 6A) and high-N-fertilizer groups (Figure 6B), and three (clusters I–III) in the combined treatment group (Figure 6C). 

In the water-deficit treatment, DAPs involved in photosynthesis and protein folding were downregulated, whereas those related to redox homeostasis, stress defense, and energy and carbohydrate metabolism were upregulated. In particular, some DAPs in clusters II–V had a high expression level at early growing stages (10–20 DPA) such as drought responsive proteins, while others in clusters I and II displayed peak expression at the late growing stage (25 DPA), mainly proteins related to carbohydrate and energy metabolism (Figure 6A). In the high-N treatment, most of the DAPs involved in photosynthesis, energy and carbohydrate metabolism, redox homeostasis, stress defense, and protein folding were upregulated at early grain development stages (Figure 6B). For example, ribulose-1,5-bisphosphate carboxylase/oxygenase small subunit (RBSC, spot 4), related to photosynthesis, was upregulated 238% at 10 DPA (Table 1). In the combined treatment, the DAPs involved in photosynthesis, which were downregulated under a water deficit, generally showed upregulated expression, such as phosphoribulokinase (PRK, spot 33), and phosphoglycerate kinase (PGK, spot 36). Furthermore, some DAPs upregulated under the high-N fertilization treatment maintained a high expression level, such as photosynthesis and energy metabolism proteins (Figure 6C).

### 2.5. Comparison of Transcription and Translation Expression Levels of Key DAPs During Grain Development

**Figure 7:**
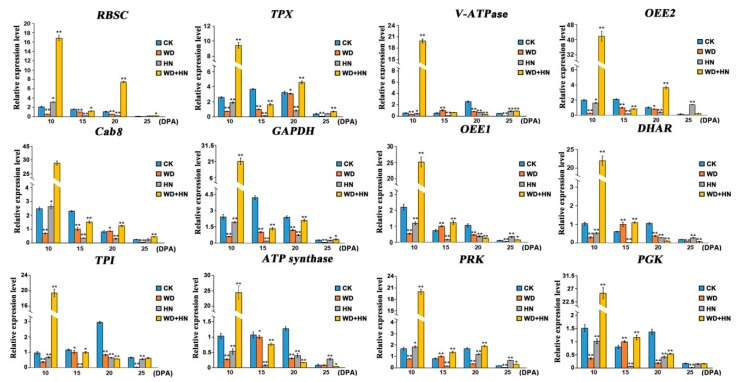
Transcription expression patterns of the genes of 12 representative differentially accumulated proteins from wheat flag leaves during grain development by quantitative real-time PCR analysis. CK: regular watering and N fertilization; WD: no watering and regular N fertilization; HN: regular watering and high-N fertilization; WD+HN: no watering and high-N fertilization; DPA, days post-anthesis. For gene abbreviations, see the main text. Significant differences compared to the control were calculated based on independent Student’s *t*-test: * p < 0.05; ** p < 0.01.

We selected 12 representative DAP genes from different functional groups to detect transcription expression profiling changes under different treatments by quantitative real-time PCR (RT-qPCR), and then compared their transcription and translation expression differences (Figure 7, Figure S2). Specific primers are provided in Table S3. In the water-deficit treatment, the expressions of the main DAP genes involved in photosynthesis, redox homeostasis, and energy and carbohydrate metabolism were downregulated, whereas several DAP genes were upregulated at 15 DPA, such as *vacuolar proton-ATPase* (*V-ATPase*, spot 8), *oxygen-evolving enhancer protein 1* (*OEE1*, spot 24), *DHAR* (spot 25), *PRK* (spot 32), and *PGK*. In the high-N treatment, the main DAP genes involved in redox homeostasis and energy and carbohydrate metabolism were downregulated, but several photosynthesis-related DAP genes were upregulated at early growth stages, including *RBSC* (spot 1), *chlorophyll a-b binding protein 8* (*Cab8*, spot 13), and *PRK* (spot 32). In the combined water-deficit and high-N treatment group, most DAP genes were upregulated at 10 DPA, followed by a downregulation at 15 DPA, excluding several redox homeostasis- and photosynthesis-related DAP genes such as *RBSC* (spot 1), *thioredoxin-dependent peroxidase* (*TPX*, spot 5), *OEE2* (spot 11), *Cab* (spot 13), and *PRK* (spot 32). As shown in Figure 7, the transcription patterns of DAP genes related to signal transduction (*V-ATPase*, spot 8) were highly consistent with its protein expression pattern, whereas four DAP genes involved in photosynthesis, redox homeostasis, energy and carbohydrate metabolism showed a poor consistency between their transcription and protein expression patterns, including *OEE1* (spot 1), *DHAR*, *ATP synthase* (spot 30), and *triosephosphate isomerase* (*TPI*, spot 26). The transcription patterns of the remaining DAP genes were consistent with their protein expression patterns under one or two treatments. For example, the transcription patterns of *TPX* under the water-deficit and high-N treatments were highly consistent with its protein expression pattern, but poor consistency was observed between its transcription pattern and protein expression pattern under the combined treatment. The disparities between transcription levels and their corresponding protein levels have been previously reported [45,46]. It may be caused by time-space span between transcription and translation, posttranscriptional modifications, as well as differential mRNA and protein degradation rates [47] 

## 3. Discussion

### 3.1. DAPs Involved in Water-Deficit Response

Water-deficit conditions caused significant physiological and proteomic changes, namely accelerated energy metabolism and grain development, and decreased grain weight and yield. Under water-deficit conditions, flag leaves yellowed at 25 DPA and showed a significant decrease in RWC and chlorophyll content (Figure 1A–D). Chlorophyll degradation and a decline in photosynthetic efficiency are indicative of senescence [48]. Furthermore, plants under drought stress showed a significant increase in MDA and soluble sugar contents (Figure 1G,H). MDA is a product of lipid peroxidation, and is often used as a proxy for the extent of oxidative damage to plant cell membranes under stress conditions [49]. Soluble sugars are important osmotic adjustment molecules that balance cellular osmotic potential and maintain physiological processes. The accumulation of soluble sugars during drought stress may improve a plant’s ability to adapt to adverse environmental conditions [50].

It is known that the inhibition of photosynthesis is one of the primary detrimental effects of water deficit due to stomatal closure and diminutions of CO_2_ uptake by leaves and intracellular CO_2_ concentration. Accordingly, a number of proteins related to photosynthesis were found with a general decreased trend under water-deficit conditions in this study, such as RuBisCO, PRK, and PGK (Table 1). The downregulation of these proteins in response to drought stress was also found in wheat leaves [9,10,29,30,31], but the abundance of RuBisCO was significantly upregulated in wheat developing grains [10,36]. Water-deficit conditions generally accelerate grain development by enhancing remobilization of pre-stored carbon reserves from vegetative organs to wheat grains [51,52]. Therefore, the rates of energy and carbohydrate metabolism in wheat flag leaves were expedited under water-deficit conditions. Proteome analysis revealed significant upregulations in key enzymes involved in energy and carbohydrate metabolism under the water-deficit treatment, such as GAPDH, TPI, aconitase, 6-phosphogluconate dehydrogenase (6-PGD, spot 38) and ATP synthase (Table 1). Among these proteins, GAPDH, TPI, and aconitase are vital enzymes for glycolysis and the tricarboxylic acid cycle, respectively, which are essential processes for carbohydrate metabolism and efficient energy production [53,54,55]. Previous studies showed that the upregulation of these three proteins was found in the drought-responsive proteome analysis of wheat leaves [9,10] and grains [34,36], but the upregulated accumulation of 6-PGD under water deficit was firstly found by the present proteome analysis, which serves as a key enzyme of the pentose phosphate pathway (PPP), an alternative pathway of glycolysis. Collectively, the downregulation of photosynthesis-related proteins and upregulation of energy and carbohydrate metabolism-related proteins were responsible for the observed significant reduction of grain weight and yield under water-deficit conditions.

Adverse environmental conditions cause imbalances in cellular redox metabolism and lead to the excessive accumulation of reactive oxygen species (ROS) and oxidative damage [56]. To alleviate oxidative damage, the higher plants have evolved oxygen-scavenging mechanisms to clear excessive ROS and maintain redox homeostasis [57,58]. Several important DAPs involved in antioxidant stress in wheat flag leaves were upregulated in response to water-deficit conditions, such as TPX, DHAR and PPO (Table 1). These results were consistent with previous proteomic analysis in wheat leaf and grain under drought stress [10,29]. TPX reduces hydrogen peroxide (H_2_O_2_) and alkyl hydroperoxides by reducing equivalents provided by thioredoxin under adverse conditions [59]. The activity of total PODs also significantly increased under drought stress (Figure 1F). H_2_O_2_ is required for the abscisic acid pathway to modulate the expression of stress-responsive genes, and could be reduced through the ascorbate–glutathione (AsA–GSH) cycle [10]. In this cycle, AsA and GSH participate in a cyclic transfer of reducing equivalents, which involves four enzymes that act to reduce H_2_O_2_ into H_2_O using electrons derived from NAD(P)H [60]. We identified one of these enzymes, DHAR, as being significantly upregulated in flag leaves under the water-deficit treatment (Table 1). During drought stress, DHAR may reduce dehydroascorbate into AsA, which is used by ascorbate peroxidase to reduce H_2_O_2_ while generating two molecules of monodehydroascorbate [61]. Therefore, the regulation of TPX and DHAR could improve plant ROS resistance during water-deficit conditions. In addition, we identified two proteolytic enzymes for the first time by proteome analysis of wheat flag leaf, ATP-dependent Clp protease proteolytic subunit (ClpP) and leucine aminopeptidase 2 (LAP2). Both proteins were significantly upregulated in water-deficit conditions. Previous studies reported that these two enzymes can function as molecular chaperone to protect protein from stress-induced damage by degrading misfolded proteins and helping proteins fold properly in stressful condition [62,63]. Therefore, we suggested that the upregulation of ClpP and LAP2 could alleviate damage from water deficit treatment. 

### 3.2. DAPs Participated in High-N Response

N is a constituent of amino acids in plants and is critical for the synthesis of proteins, chlorophyll, and numerous other metabolites [64]. During reallocation of N assimilation, large amounts of N are invested into building photosynthetic complexes [19]. In contrast to the water-deficit treatment, high-N fertilization significantly enhanced chlorophyll content and Pn and increased grain weight and yield by upregulating photosynthesis-, energy and carbohydrate metabolism-related proteins. These results were consistent with previous reports that application of high-N fertilizer resulted in significant increases in leaf length and area, chlorophyll content and grain yield [42]. Photosynthesis-related proteins such as RuBisCO, PRK, and PGK were significantly upregulated under this treatment (Table 1). These changes of RuBisCO and PRK were also found in proteome analysis of wheat grains under high-N treatment [43]. The upregulation of these enzymes likely enhanced the rate of photosynthesis, thereby providing more photoassimilates for grain filling, leading to an improvement in grain weight. High N application has been shown to significantly increase the protein content in wheat grains, mainly by promoting protein synthesis [44,65,66]. In this study, we firstly found significant upregulation of peptidyl-prolyl *cis*-trans isomerase (PPIase) under high-N fertilization (Table 1). PPIase accelerates protein folding by catalyzing the *cis*–trans isomerization of imidic proline peptide bonds in oligopeptides [67].

Inorganic N assimilation is a highly energy-intensive process, requiring substantial ATP and NAD(P)H [47]. We identified several upregulated DAPs associated with ATP synthesis and NAD(P)H production in the high-N treatment, including ATP synthase, GAPDH, TPI, FBA (spot 37), 6-PGD, and aconitase. ATP synthase is a ubiquitous membrane enzyme that plays a key role in biological energy metabolism, which mainly functions in ATP synthesis from adenosine diphosphate (ADP) and inorganic phosphate (Pi). In the previous proteome analyses, the alpha and beta subunits of ATP synthase were respectively identified in wheat seedling leaf and grain with a significant upregulation under high-N treatment [39,41,43]. Among these enzymes, GAPDH, FBA, and 6-PGD were upregulated in the high-N treatment (Table 1), and they are key enzymes in carbohydrate metabolism pathways, such as glycolysis (GAPDH and FBA) and the pentose phosphate pathway (6-PGD). Interestingly, the upregulated accumulation of FBA, TPI, and GAPDH enzymes were also found in seedling leaves of wheat plants grown in half-strength nutrient solution with high-N level [39]. Furthermore, both FBA and GAPDH were phosphorylated in the developing wheat grains under high-N fertilization, and their phosphorylation may benefit by regulating enzyme activities and enhance carbohydrate metabolism [42]. Due to the enhanced photosynthesis rate under the high-N treatment, more photoassimilates were produced and accumulated in wheat leaves and stems. N fertilization may increase the remobilization efficiency of accumulated photoassimilates during grain filling, thereby increasing grain weight [68]. 

### 3.3. DAPs Responsive to Combining Water-Deficit and High-N Treatments 

The combined treatment (water deficit and high-N fertilization) had significant effects on the physiological characteristics and proteome composition of wheat flag leaves, including increased chlorophyll content and Pn in the early grain-filling periods (Figure 1C,E), increased grain weight and yield, and the upregulation of photosynthesis- and carbohydrate metabolism-related proteins and oxidation stress-responsive proteins. These results are consistent with recent findings that water stress can substantially accelerate grain filling, and shorten the grain-filling period, under both normal and high-N fertilizer applications [69]. We found upregulation of three DAPs involved in photosynthesis under the combined treatment, including the RuBisCO large subunit, PRK, and PGK, which showed downregulation and upregulation under water-deficit and high-N treatments, respectively (Table 1). This suggests that high-N fertilization can increase the accumulation of photosynthetic products under water-deficit conditions by enhancing the photosynthetic capacity of wheat flag leaves, particularly at the early grain-filling stages. Significantly, previous genome-wide transcriptional profiling analysis showed that the period from 11–15 DPA was more critical than the 15–20 DPA stage for the synthesis and accumulation of nutritive reserves [70]. In addition, two DAPs involved in energy and carbohydrate metabolism (FBA and GAPDH) upregulated in the combined treatment were also upregulated in the high-N treatment (Table 1). Therefore, high-N fertilization could offset the losses in grain weight and yield caused by water-deficit through enhancing energy and carbon metabolism and promoting remobilization and transfer of accumulated photoassimilates from flag leaves to grains.

### 3.4. A Putative Synergistic Response Network Under Water-Deficit and High-N Conditions

**Figure 8:**
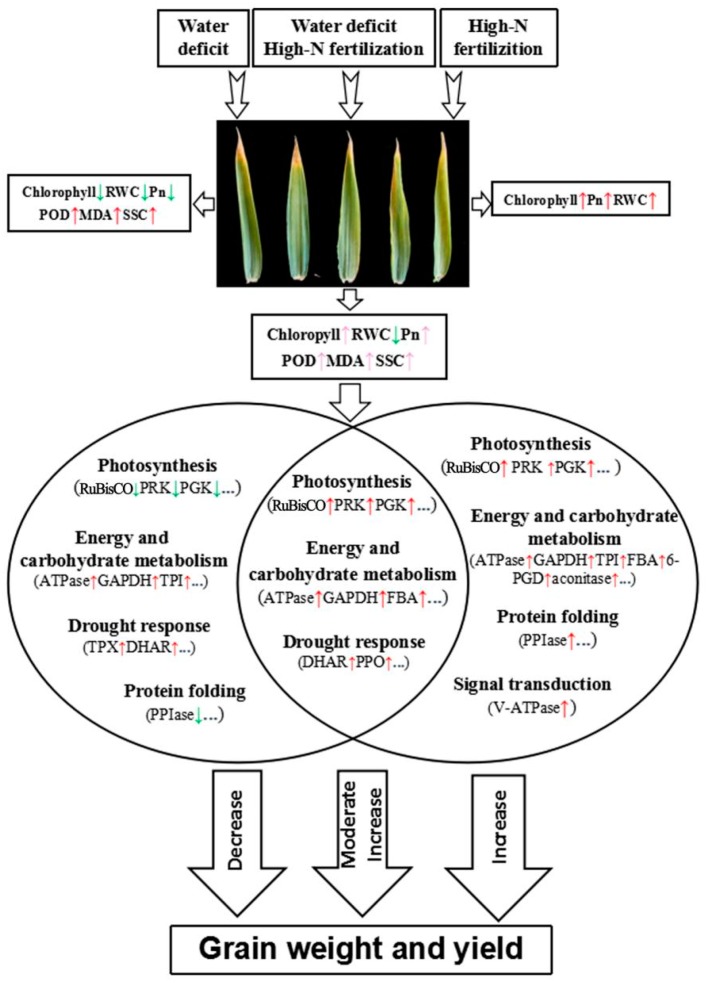
A putative synergistic response network in flag leaf proteomes to water-deficit and high-N conditions. RWC, relative water content; Pn, net photosynthetic rate; SSC, soluble sugar content; RuBisCO, ribulose-1,5-bisphosphate. carboxylase/oxygenase; PRK, phosphoribulokinase; PGK, phosphoglycerate kinase; TPX, thioredoxin-dependent peroxidase; DHAR, dehydroascorbate reductase; GAPDH, glyceraldehyde 3-phosphate dehydrogenase; TPI, triosephosphate isomerase; FBA, fructose-1,6-bisphosphate aldolase; 6-PGD, 6-phosphogluconate dehydrogenase; PPIase, peptidyl-prolyl cis–trans isomerase. The red arrow represents upregulation, pink and green arrows represent moderate upregulation and downregulation, respectively.

We propose a putative synergistic response network in wheat flag leaf proteomes to water-deficit and high-N conditions (Figure 8). When subjected to water-deficit and/or high-N fertilization, a series of physiological changes occurred in flag leaves, including a significant reduction in RWC, total chlorophyll content, Pn caused by drought stress, and a significant increase in these same parameters under high-N application. A moderate promotion of these parameters was observed under the combined treatment. Stress-related parameters, MDA, POD activity, and soluble sugar content were increased under the water-deficit and combined treatments. The main DAPs involved in photosynthesis and protein folding were upregulated and downregulated under water-deficit and high-N conditions, respectively. In the combined treatment, DAPs associated with photosynthesis and energy and carbohydrate metabolism showed a moderate upregulation. Ultimately, these physiological and proteomic synergistic responses of wheat flag leaves led to significant changes in grain weight and yield among the water-deficit, high-N, and combined treatments. 

## 4. Materials and Methods

### 4.1. Wheat Materials, Field Trials, and Sampling 

Chinese elite winter wheat cultivar “Jingdong 17” (*Triticum aestivum* L., 2n = 6X = 42, AABBDD) was used as material. Field trials were performed in the experimental station of China Agricultural University, Wuqiao, Hebei Province (116°37′23″ E, 37°16′02″ N) during the 2017–2018 wheat growing season. The soil type of experimental field was clay-loam soil. The soil bulk density and field capacity in 0–200 cm soil layers with 20 cm increment are presented in Table S4. The topsoil 0–40 cm nutrient contents contained 14.2 g/kg of organic matter, 1.02 g/kg of total nitrogen, 18.4 mg/kg of available phosphorus, and 102.8 mg/kg of available potassium. The monthly rainfall and daily mean temperature during 2017–2018 wheat growing season were shown in Figure S3. 

According to a previous report, the biomass of wheat population and population spikelets increased with N application amount in the range of 0–240 kg/hm^2^ of fertilizer urea (NH_2_)_2_CO [24]. In this study, 180 and 240 kg/hm^2^ urea was used as normal N fertilization and high-N fertilization, respectively. The field experiments included four treatments: a control group with well-watered irrigation and normal N fertilization (CK, control treatment), well-watered irrigation with high-N fertilization (HN, high-N fertilizer treatment), no irrigation with normal N fertilization (WD, water-deficit treatment), and no irrigation with high-N fertilization (WD+HN, water-deficit treatment and high-N fertilizer). The well-watered irrigation was subjected to traditional flood irrigation with 75 mm at both jointing and flowering stages, while no irrigation was set as rain-fed region without artificial water irrigation. The N fertilization application was conducted as follows: 120 kg/hm^2^ (sowing time) and 60 kg/hm^2^ (jointing stage) for CK, 120 kg/hm^2^ (sowing time) and 120 kg/hm^2^ (jointing stage) for high-N treatment. To avoid burning seedlings caused by fertilization under drought condition at the jointing stage, N fertilization of 180 and 240 kg/hm^2^ was applied at sowing time for drought and combined treatment, respectively. Each treatment was conducted with three biological duplications. Each experimental plot was 4 m × 9 m with row spaced at 0.16 m. One meter wide zone between plots was designed as an unirrigated zone to minimize the interference from adjacent plots. The plants were marked after flowering, and flag leaves from four periods (10, 15, 20 and 25 day post-anthesis, DPA) in three biological replicates were collected, and immediately transferred in liquid nitrogen, and then stored at −80 °C prior to analysis. 

### 4.2. Measurements of Flag Leaf Physiological Parameters and Grain Yield

The relative water content (RWC), malondialdehycle (MDA) content, peroxidase (POD) activity, soluble sugar, and total chlorophyll content of flag leaves from all treatments in three biological replicates were measured at 10, 15, 20, and 25 DPA according to the method described by [71]. The net photosynthesis rate (Pn) of flag leaves at 10, 15, 20, and 25 DPA was measured by using an LI-6400XT Portable Photosynthesis System (LI-COR Bioscience, Inc., Lincoln, NE, USA) with 6400-40 Leaf Chamber Fluorometer under artificial light (actual intensity at 1200 ± 50 µmol·m^−2^·s^−1^). The instrument was calibrated for humidity at 20–30% relative humidity and the setting of chamber CO_2_ concentration was 400 μL·L^−1^ with a flow rate of 500 μmol·s^−1^. Grain yield (with 13% water content) were measured from an area of 4 m^2^ in each plot at maturity. The thousand grain weight (TGW) was calculated by weighing 1000 seeds from each sample and averaged from three replicates. Statistical analyses were conducted by using independent student’s *t*-tests with SPSS statistics software (Version 19.0). 

### 4.3. Protein Extraction, 2-DE, and Image Analysis 

Total proteins of wheat flag leaves from 10, 15, 20, and 25 DPA in three biological replicates were extracted according to [72]. Two-dimensional electrophoresis (2-DE) was used to separate the differentially accumulated protein (DAP) spots based on [73]. After 2-DE electrophoresis, all gels were stained with Coomassie Brilliant Blue (R-250:G-250 = 4:1). The ImageMaster 2D Platinum Software Version 7.0 (GE Healthcare Bio-Sciences AB, Uppsala, Sweden) was used to analyze the gel images, and the spots with significant and biological reproducible changes (protein abundance variation at least 2-fold, Student’s *t*-test, *p* < 0.05) were considered as DAP spots. 

### 4.4. Protein Identification Using MALDI-TOF/TOF-MS 

The DAP spots were excised from preparative 2-DE gels manually, and then digested with trypsin for further identification using matrix-assisted laser desorption/ionization time-of-flight/time-of-flight mass spectrometry (MALDI-TOF/TOF-MS). The MS/MS spectra were obtained using an ABI 4800 Proteomics Analyzer MALDI-TOF/TOF (Applied Biosystems, Foster City, CA) operating in a result-dependent acquisition mode and searched in the National Center for Biotechnology Information (NCBI) non-redundant wheat database using MASCOT version 2.1 (Matrix Science, London, UK) with the following parameter settings: trypsin cleavage; one missed cleavage allowed; carbamidomethylation as fixed modification; oxidation of methionines and phosphorylation of serine, threonine, and tyrosine allowed as variable modifications; peptide mass tolerance of 100 ppm; and fragment tolerance of −0.3 Da. All searches were evaluated based on the significant scores obtained from MASCOT. Both total ion score confidence interval percentage and protein score confidence interval percentage were set above 95% and the significance threshold *p* < 0.05 was used for MS/MS data analysis. 

### 4.5. Bioinformatic Analysis

Venn diagram analysis of the identified DAP spots was conducted by using an online tool (http://bioinformatics.psb.ugent.be/webtools/Venn/). The protein function classification was performed based on the functional annotation searched against UniProt database (https://www.uniprot.org/). The sub-cellular localization of the identified proteins was predicated according to the combination of predicated results from WoLF PSORT (https://wolfpsort.hgc.jp/), Plant-mPLoc (http://www.csbio.sjtu.edu.cn/bioinf/plant-multi/), CELLO v.2.5 (http://cello.life.nctu.edu.tw/) and UniProtKB (https://www.uniprot.org/help/uniprotkb/). Protein accumulation clustering analysis of DAP spots was conducted by using Cluster software version 3.0. Euclidean distance with a set of Ward’s criteria. Cluster results were visualized by using Java TreeView software version 1.1.6r4. 

### 4.6. Acquisition of the DAP Genes and Subcellular Localization

The acquisition of the expected DAP genes was based on [74]. The sequences of the selected DAPs were used as a tBLASTn query against the wheat EST database in NCBI, all matching ESTs were assembled to get a unigene by using CAP3 software, and then the specific primers (Table S2) were designed to amplificate the full-length cDNA. 

For subcellular localization assay, the full-length coding sequences were cloned and then ligated into 16318hGFP vector, which encodes GFP under the constitutive control of the cauliflower mosaic virus 35S promoter. Isolation and PEG-mediated transformation of wheat protoplasts were performed according to [75]. Subsequently, the transformed protoplasts were collected and resuspended with protoplast culture medium. After 72 h of incubation at 23 °C in darkness condition, the green fluorescence and chlorophyll red auto-fluorescence were monitored with confocal laser scanning microscope (Leica TCS SP5, Wetzlar, Germany). 

### 4.7. mRNA Extraction, cDNA Synthesis and RT-qPCR 

The quantitative real time polymerase chain reaction (RT-qPCR) were conducted to determine the dynamic transcript expression levels of the DAP genes. Total RNA of flag leaves from four grain developmental periods (10, 15, 20, and 25 DPA) was extracted using TRIZOL Reagent (Invitrogen, Breda, Netherlands) according to the manufacturer’s instructions. Genomic DNA was removed by digesting each sample (20–50 μg of total RNA) with DNase I (Promega, Madison, Wisconsin, USA). Then reverse transcription reactions were performed with the PrimeScript^®^ RT Reagent Kit with gDNA Eraser (TaKaRa, Shiga, Japan) according to the manufacturer’s instructions. Gene-specific qPCR-primers were designed by using online tool Primer3Plus (http://www.primer3plus.com/) and primer specificity was confirmed with melting curve analysis of RT-qPCR products. Ubiquitin gene was used as reference for normalization. The sample mixture of RT-qPCR was prepared according to the procedures reported by [76] and each sample was conducted with three biological replicates. The relative expression levels were detected using the comparative threshold cycle method 2^−^^△△*C*t^ [77]. 

## 5. Conclusions

Water-deficit conditions dramatically suppressed the Pn of wheat flag leaves and shortened the grain-filling period, leading to a significant decrease in grain weight and yield. In contrast, high-N fertilization enhanced Pn and grain-filling, leading to an increase in grain weight and yield. High-N fertilization could offset losses in grain weight caused by drought by enhancing the photosynthetic ability of flag leaves in early grain-filling stages. Flag leaf proteome analysis revealed that the DAPs related to photosynthesis and protein folding were downregulated and upregulated under water-deficit and high-N treatments, respectively. The combined treatment induced a moderate upregulation in DAPs involved in photosynthesis and energy and carbohydrate metabolism. Our results provide new insight into plant proteome response mechanisms under water-deficit conditions and high-N fertilization.

## Figures and Tables

**Table 1:** The representative DAPs of wheat flag leaves identified by MALDI-TOF/TOF-MS under water-deficit (WD), high-N fertilizer and combined (CB) treatments.

Spot No.	Protein Name	Water-Deficit Treatment (10/15/20/25 DPA)	High-N Fertilizer Treatment (10/15/20/25 DPA)	Combined Treatment (10/15/20/25 DPA)
**Signal transduction**				
8	Vacuolar proton-ATPase	−25%/+22%/−2%/+1%	−17%/−4%/−11%/+81%	+38%/−12%/+14%/+105%
**Redox homeostasis and stress defense**				
5	Thioredoxin-dependent peroxidase	−47%/+9%/+11%/+111%	−20%/+24%/−16%/+112%	+36%/+9%/+3%/+24%
7	Germin-like protein	−18%/+81%/+101%/−24%	−7%/−20%/−24%/+15%	−4%/+103%/−49%/−12%
25	Dehydroascorbate reductase	+41%/−59%/+112%/+12%	+32%/+12%/−100%/−59%	−3%/−15%/+101%/+31%
52	Polyphenol oxidase	+63%/+21%/−15%/+27%	+17%/+18%/−15%/−100%	+43%/+113%/−100%/+32%
**Carbohydrate metabolism**				
34	Glyceraldehyde-3-phosphate dehydrogenase B	−36%/−47%/-40%/+133%	−21%/−40%/−26%/+118%	−34%/−33%/−21%/+89%
26	Triosephosphate isomerase	−37%/−6%/+108%/+42%	−69%/+101%/−100%/−100%	−47%/−23%/+36%/+28%
37	Fructose-1,6-bisphosphate aldolase	+21%/+18%/+59%/−87%	−45%/+151%/+19%/−43%	−35%/+102%/−69%/+115%
38	6-phosphogluconate dehydrogenase	+134%/−38%/+67%/+33%	+153%/−24%/−8%/−35%	+76%/+2%/+74%/−69%
55	Aconitase	+130%/−41%/−29%/+133%	+13%/−59%/−77%/+53%	+43%/−55%/−13%/+35%
**Energy metabolism**				
30	ATP synthase subunit	+80%/−15%/−21%/+54%	+121%/+37%/+73%/+118%	−19%/−28%/−22%/+14%
47	ATP synthase CF1 alpha subunit	+36%/−20%/−38%/+3%	+109%/−46%/−75%/+89%	−14%/−45%/−37%/+128%
48	ATP synthase CF1 alpha subunit	−5%/−39%/+30%/−8%	+76%/−32%/+64%/+54%	+34%/+43%/+4%/−5%
53	ATP synthase CF1 alpha subunit	+101%/−22%/+11%/−14%	+122%/−24%/−54%/+35%	+18%/−29%/−49%/+2%
44	ATP synthase CF1 alpha subunit	+121%/−48%/−11%/+97%	+104%/+54%/−62%/+35%	+20%/−54%/−35%/+23%
45	ATP synthase CF1 beta subunit	−4%/−5%/−42%/−33%	−14%/+20%/−41%/−20%	−76%/+7%/+3%/+27%
**Photosynthesis**				
1	Ribulose-1,5-bisphosphate carboxylase/oxygenase small subunit	−21%/+52%/−59%/+1%	−16%/+38%/−26%/+4%	+35%/−12%/−1%/+4%
4	Ribulose-1,5-bisphosphate carboxylase/oxygenase small subunit	−64%/−63%/+24%/−16%	+238%/+14%/−20%/−35%	+17%/−13%/+50%/−38%
23	Ribulose-1,5-bisphosphate carboxylase/oxygenase small subunit	+119%/−45%/−89%/+1%	+130%/+13%/−100%/−11%	+54%/−51%/−100%/−34%
40	Ribulose-1,5-bisphosphate carboxylase/oxygenase large subunit	+67%/−13%/−52%/−55%	+119%/+4%/−10%/−30%	−4%/−20%/+43%/−37%
41	Ribulose-1,5-bisphosphate carboxylase/oxygenase large subunit	−20%/+82%/+8%/−25%	−35%/−18%/+5%/+110%	−46%/+101%/+111%/−12%
60	Ribulose-1,5-bisphosphate carboxylase/oxygenase large subunit	+26%/−18%/+33%/−1%	+8%/−68%/−20%/+101%	+7%/−49%/−27%/−8%
11	Oxygen-evolving enhancer protein 2, chloroplastic	+35%/−0.11%/−8%/+111%	+15%/−20%/−36%/+63%	+10%/−14%/−11%/+140%
12	23kDa oxygen evolving protein of photosystem II	−52%/−35%/+74%/+48%	−61%/+12%/−45%/+5%	−45%/+1%/+20%/−54%
24	Oxygen-evolving enhancer protein 1	+5%/+1%/+131%/−70%	-40%/−41%/−22%/+4%	+65%/−59%/+28%/+2%
32	Phosphoribulokinase	−12%/−13%/−100%/+31%	−28%/+102%/−22%/−37%	−15%/−3%/−26%/+52%
33	Phosphoribulokinase	+41%/+81%/−86%/-62%	-45%/+27%/+102%/-89%	−23%/+65%/+111%/−33%
36	Phosphoglycerate kinase	−59%/+8%/−23%/+48%	−12%/+9%/−19%/+115%	−36%/+1%/−25%/+101%
13	Chlorophyll a-b binding protein 8	−18%/−19%/+26%/+309%	−36%/+10%/−31%/+134%	+3%/+8%/−4%/+35%
14	Chlorophyll a-b binding protein 8	+6%/+111%/−13%/−12%	+29%/+46%/+17%/−14%	−30%/+15%/+12%/+10%
29	Photosystem II stability/assembly factor HCF136	+40%/−11%/−1%/−89%	+12%/+11%/0%/−13%	+14%/−23%/+63%/+84%
54	NADP-dependent malic enzyme	−84%/−68%/+33%/+31%	−26%/−2%/+394%/+1%	−43%/−48%/+21%/+101%
**Amino acid metabolism and protein folding**				
2	Putative glycine decarboxylase subunit	−16%/+137%/−1%/+26%	−4%/+133%/−19%/−10%	+41%/+16%/−26%/+41%
43	Leucine aminopeptidase 2	−27%/+12%/+64%/−18%	+24%/+28%/−48%/−79%	−49%/+27%/+116%/−11%
9	Peptidyl-prolyl cis-trans isomerase 2	−32%/−52%/+28%/+66%	−7%/−19%/+1%/+101%	+6%/−15%/+74%/+52%
15	ATP-dependent Clp protease proteolytic subunit	+41%/+101%/−53%/−52%	−49%/+24%/+13%/−75%	−67%/−18%/+21%/+14%
57	ATP-dependent Clp protease ATP-binding subunit clpA-like protein	+238%/−12%/−21%/+67%	+215%/+21%/−66%/+58%	+26%/−8%/−61%/+19%

## Supplementary Materials

The following are available online at https://www.mdpi.com/1422-0067/21/6/2098/s1. Figure S1. Two-dimensional electrophoresis (2-DE) maps of wheat flag leaf proteome under water-deficit, high-N fertilizer and their combined treatments. Figure S2. Translation expression patterns of 12 representative DAP genes from wheat flag leaves during grain development. Figure S3. The monthly rainfall and daily mean temperature during 2017–2018 wheat growing season. Table S1. The DAPs from wheat flag leaf identified by MALDI-TOF/TOF-MS. Table S2. List of primers used for gene clone and vector construction. Table S3. List of primers used for RT-qPCR. Table S4. The soil bulk density and field capacity in 0–200 cm soil layers with 20 cm increment in the experimental field.

## Abbreviations

DAP	Differentially accumulated protein
DPA	Day post-anthesis
RWC	Relative water content
MDA	Malondialdehyde
TGW	Thousand grain weight
Pn	Net photosynthesis efficiency
2-DE	Two-dimensional gel electrophoresis
GFP	Green fluorescent protein
GAPDH	Glyceraldehyde 3-phosphate dehydrogenase
DHAR	Dehydroascorbate reductase
BAS1	2-Cys peroxiredoxin BAS1
PPO	Polyphenol oxidase
FBA	Fructose-1,6-bisphosphate aldolase
RBSC	Ribulose-1,5-bisphosphate carboxylase/oxygenase small subunit
PRK	Phosphoribulokinase
PGK	Phosphoglycerate kinase
RT-qPCR	Quantitative real time polymerase chain reaction
V-ATPase	Vacuolar proton-ATPase
OEE1	Oxygen-evolving enhancer protein 1
Cab8	Chlorophyll a-b binding protein 8
TPX	Thioredoxin-dependent peroxidase
TPI	Triosephosphate isomerase
PPIase	Peptidyl-prolyl cis–trans isomerase
6-PGD	6-phosphogluconate dehydrogenase
NCBI	National Center for Biotechnology Information

## References

[1] Shewry P.R. (2009). Wheat. J. Exp. Bot..

[2] Wim V., Edoardo B., Stefanie D.B., Klaas V., Marlies D., Mario E.P., Inzé D. (2013). Molecular and Physiological Analysis of Growth-Limiting Drought Stress in Brachypodium distachyon Leaves. Mol. Plant.

[3] Godfray H.C.J., Beddington J.R., Crute I.R., Haddad L., Lawrence D. (2010). Food security: The challenge of feeding 9 billion people. Science.

[4] Tilman D., Balzer C., Hill J., Befort B.L. (2011). Global food demand and the sustainable intensification of agriculture. Proc. Natl. Acad. Sci. USA.

[5] Lesk C., Rowhani P., Ramankutty N. (2016). Influence of extreme weather disasters on global crop production. Nature.

[6] Zipper S.C., Qiu J., Kucharik C.J. (2016). Drought effects on US maize and soybean production: Spatiotemporal patterns and historical changes. Environ. Res. Lett..

[7] Matiu M., Ankerst D.P., Menzel A. (2017). Interactions between temperature and drought in global and regional crop yield variability during 1961–2014. PLoS ONE.

[8] IPCC (2007). Summary for Policy Makers. Climate Change 2007: The Physical Science Basis. Contribution of Working Group I to the Fourth Assessment Report.

[9] Michaletti A., Naghavi M.R., Toorchi M., Zolla L., Rinalducci S. (2018). Metabolomics and proteomics reveal drought-stress responses of leaf tissues from spring-wheat. Sci. Rep..

[10] Deng X., Liu Y., Xu X.X., Liu D.M., Zhu G.R., Yan X., Wang Z.M., Yan Y.M. (2018). Comparative proteome analysis of wheat flag leaves and developing grains under water deficit. Front. Plant Sci..

[11] Anjum S.A., Farooq M., Wang L.C., Xue L.L., Wang S.G., Wang L., Zhang S., Chen M. (2011). Gas exchange and chlorophyll synthesis of maize cultivars are enhanced by exogenously-applied glycinebetaine under drought conditions. Plant Soil Environ..

[12] Nezhadahmadi A., Prodhan Z.H., Faruq G. (2013). Drought Tolerance in Wheat. Sci. World J..

[13] Foyer C.H., Noctor G., Verrier P., Plaxton W., McManus M.T. (2006). Annual Plant Reviews: Control of Primary Metabolism in Plants.

[14] Dreccer M.F., Oijen M.V., Schapendonk A.H.C.M., Rabbinge C.S. (2000). Dynamics of vertical leaf nitrogen distribution in a vegetative wheat canopy. Impact on canopy photosynthesis. Ann. Bot..

[15] Javier G., Jaume F., Maurici M.U.S., Josep C., Elkadri L., Hipolito M. (2003). Relationship between maximum leaf photosynthesis, nitrogen content and specific leaf area in balearic endemic and non-endemic Mediterranean species. Ann. Bot..

[16] Tambussi E.A., Nogues S., Ferrio P., Voltas J., Araus J.L. (2005). Does higher yield potential improve barley performance in Mediterranean conditions?: A case study. Field Crops Res..

[17] Slafer G.A., Andrade F.H., Storre E.H. (1990). Genetic improvement effects on pre-anthesis physiological attributes related to wheat grain yield. Field Crops Res..

[18] Makino A., Osmond B. (1991). Solubilization of ribulose-1,5-bisphosphate carboxylase from the membrane fraction of pea leaves. Photosynth. Res..

[19] Zhu X.G., Long S.P., Ort D.R. (2008). What is the maximum efficiency with which photosynthesis can convert solar energy into biomass?. Curr. Opin. Biotechnol..

[20] Zelitch I. (1982). The Close Relationship Between Net Photosynthesis and Crop Yield. BioScience.

[21] Erisman J.W. (2004). The Nanjing declaration on management of reactive nitrogen. Bioscience.

[22] Li S.X., Wang Z.H., Hu T.T., Gao Y.J., Stewart B.A., Sparks D.L. (2009). Nitrogen in dryland soils of china and its management. Advances in Agronomy.

[23] Malhi S.S., Grant C.A., Johnston A.M., Gill K.S. (2001). Nitrogen fertilization management for no-till cereal production in the Canadian Great Plains: A review. Soil Tillage Res..

[24] Li F.J., Xu X.X., Xiao Y.G., He Z.H., Wang Z.M. (2016). Effect of nitrogen on yield related traits and nitrogen utilization efficiency in Zhongmai 175 and Jingdong 17. Acta Agron. Sin..

[25] Yao N., Li Y., Li N., Yang D.Q., Ayantobo O.O. (2018). Bias correction of precipitation data and its effects on aridity and drought assessment in China over 1961–2015. Sci. Total Environ..

[26] Chen Y., Li W., Deng H., Fang G., Li Z. (2016). Changes in Central Asia’s water tower: Past, present and future. Sci. Rep..

[27] Wijk K.J.V. (2001). Challenges and Prospects of Plant Proteomics. Plant. Physiol..

[28] Peng Z.Y., Wang M.C., Li F., Lv H.J., Li C.L., Xia G.M. (2009). A Proteomic Study of the Response to Salinity and Drought Stress in an Introgression Strain of Bread Wheat. Mol. Cell. Proteom..

[29] Ford K.L., Cassin A., Bacic A. (2011). Quantitative proteomic analysis of wheat cultivars with differing drought stress tolerance. Front. Plant Sci..

[30] Budak H., Akpinar B.A., Unver T., Turktas M. (2013). Proteome changes in wild and modern wheat leaves upon drought stress by two-dimensional electrophoresis and nanoLC-ESI-MS/MS. Plant Mol. Biol..

[31] Ye J.X., Wang S.P., Zhang F.J., Xie D.Q., Yao Y.H. (2013). Proteomic analysis of leaves of different wheat genotypes subjected to PEG 6000 stress and rewatering. Plant Omics.

[32] Hao P.C., Zhu J.T., Gu A.Q., Lv D.W., Ge P., Chen G.X., Li X.H., Yan Y.M. (2014). An integrative proteome analysis of different seedling organs in tolerant and sensitive wheat cultivars under drought stress and recovery. Proteomics.

[33] Zhang H.M., Zhang L.S., Lv H., Yu Z.Y., Zhang D.P., Zhu W.N. (2014). Identification of changes in *Triticum aestivum* L. leaf proteome in response to drought stress by 2D-PAGE and MALDI-TOF/TOF mass spectrometry. Acta Physiol. Plant..

[34] Faghani E., Gharechahi J., Komatsu S., Mirzaei M., Khavarinejad R.A., Najafi F., Farsad L.K., Salekdeh G.H. (2015). Comparative physiology and proteomic analysis of two wheat genotypes contrasting in drought tolerance. J. Proteom..

[35] Jiang S.S., Liang X.N., Li X., Wang S.L., Lv D.W., Ma C.Y., Li X.H., Ma W.J., Yan Y.M. (2012). Wheat Drought-Responsive Grain Proteome Analysis by Linear and Nonlinear 2-DE and MALDI-TOF Mass Spectrometry. Int. J. Mol. Sci..

[36] Ge P., Ma C.Y., Wang S.L., Guo G.F., Ma W.J., Yan Y.M. (2012). Comparative proteomic analysis of grain development in two spring wheat varieties under drought stress. Anal. Bioanal. Chem..

[37] Nadaud I., Girousse C., Debiton C., Chambon C., Bouzidi M.F., Martre P., Branlard G. (2010). Proteomic and morphological analysis of early stages of wheat grain development. Proteomics.

[38] Zhang Y.F., Huang X.W., Wang L.L., Wei L., Wu Z.H., You M.S., Li B.Y. (2014). Proteomic Analysis of Wheat Seed in Response to Drought stress. J. Integr. Agric..

[39] Chandna R., Ahmad A. (2015). Nitrogen stress-induced alterations in the leaf proteome of two wheat varieties grown at different nitrogen levels. Physiol. Mol. Biol. Plants.

[40] Bahrman N., Gouy A., Devienne-Barret F., Hirel B., Vedele F., Gouis J.L. (2005). Differential change in root protein patterns of two wheat varieties under high and low nitrogen nutrition levels. Plant Sci..

[41] Bahrman N., Gouis J.L., Negroni L., Amilhat L., Leroy P., Lainé A.L., Jaminon O. (2004). Differential protein expression assessed by two-dimensional gel electrophoresis for two wheat varieties grown at four nitrogen levels. Proteomics.

[42] Zhen S.M., Deng X., Zhang M., Zhu G.R., Lv D.W., Wang Y.P., Zhu D., Yan Y.M. (2017). Comparative phosphoproteomic analysis under high-nitrogen fertilizer reveals central phosphoproteins promoting wheat grain starch and protein synthesis. Front. Plant Sci..

[43] Zhen S.M., Deng X., Li M.F., Zhu D., Yan Y.M. (2018). 2D-DIGE comparative proteomic analysis of developing wheat grains under high-nitrogen fertilization revealed key differentially accumulated proteins that promote storage protein and starch biosynthesis. Anal. Bioanal. Chem..

[44] Araus J.L., Tapia L. (1987). Photosynthetic Gas Exchange Characteristics of Wheat Flag Leaf Blades and Sheaths during Grain Filling: The Case of a Spring Crop Grown under Mediterranean Climate Conditions. Plant Physiol..

[45] Bian Y.W., Deng X., Yan X., Zhou J.X., Yuan L.L., Yan Y.M. (2017). Integrated proteomic analysis of Brachypodium distachyon roots and leaves reveals a synergistic network in the response to drought stress and recovery. Sci. Rep..

[46] Chen Z.Y., Zhu D., Wu J.S., Cheng Z.W., Yan X., Deng X., Yan Y.M. (2018). Identification of differentially accumulated proteins involved in regulating independent and combined osmosis and cadmium stress response in Brachypodium seedling roots. Sci. Rep..

[47] Torabi S., Wissuwa M., Heidari M., Naghavi M.R. (2009). Comparative proteome approach to decipher the mechanism of rice adaptation to phosphorous deficiency. Proteomics.

[48] Munne-Bosch S., Alegre L. (2002). The function of tocopherols and toctrienols in plants. Crit. Rev. Plant Sci..

[49] Agafonova N.V., Doronina N.Y., Trotsenko Y.A. (2016). Enhanced Resistance of Pea Plants to Oxidative: Stress Caused by Paraquat during Colonization by Aerobic Methylobacteria. Prikl. Biokhim. Mikrobiol..

[50] Yu Y.H., Bi C.X., Wang Q., Ni Z.Y. (2019). Overexpression of TaSIM provides increased drought stress tolerance in transgenic Arabidopsis. Biochem. Biophys. Res. Commun..

[51] Yang J.H., Zhang J.H., Huang Z.L., Zhu Q.S., Wang L. (2000). Remobilization of carbon reserves is improved by controlled soil-drying during grain filling of wheat. Crop. Sci..

[52] Yang J.C., Zhang J.H., Wang Z.Q., Zhu Q.S., Liu L.J. (2001). Water deficit-induced senescence and its relationship to the remobilization of pre-stored carbon in wheat during grain filling. Agron. J..

[53] Beinert H., Kennedy M.C., Stout C.D. (1996). Aconitase as Iron−Sulfur Protein, Enzyme, and Iron-Regulatory Protein. Chem. Rev..

[54] Plaxton W.C. (2001). The organization and regulation of plant glycolysis. Annu. Rev. Plant Physiol. Plant Mol. Biol..

[55] Flint D.H., Allen R.M. (1996). Iron-Sulfur Proteins with Nonredox Functions. Chem. Rev..

[56] Cheng Z.W., Dong K., Ge P., Bian Y.W., Dong L.W., Deng X., Li X.H., Yan Y.M. (2015). Identification of Leaf proteins differentially accumulated between wheat cultivars distinct in their levels of drought tolerance. PLoS ONE.

[57] Zhang J., Kirkham M.B. (1994). Drought-stress-induced changes in activities of superoxide dismutase, catalase, and peroxidase in wheat species. Plant Cell Physiol..

[58] Drazkiewicz M., Skórzynska-Polit E., Krupa Z. (2007). The redox state and activity of superoxide dismutase classes in Arabidopsis thaliana under cadmium or copper stress. Chemosphere.

[59] Aymeric G., Camilla H., Myroslawa M.M., Uwe K., Pierre L.M., Jean-Pierre J., Paulette D. (2002). Isolation and characterization of a thioredoxin-dependent peroxidase from Chlamydomonas reinhardtii. Eur. J. Biochem..

[60] Noctor G., Foyer C.H. (1998). Ascorbate and glutathione: Keeping active oxygen under control. Annu. Rev. Plant Physiol. Plant Mol. Biol..

[61] Eltayeb A.E., Kawano N., Badawi G.H., Kaminaka H., Sanekata T., Morishima I., Shibahara T., Inanaga S., Tanaka K. (2006). Enhanced tolerance to ozoneand drought stresses in transgenic tobacco overexpressing dehydroascorbate reductase in cytosol. Physiol. Plant..

[62] Halperin T., Zheng B., Itzhaki H., Clarke A.K., Adam Z. (2001). Plant mitochondria contain proteolytic and regulatory subunits of the ATP-dependent Clp protease. Plant Mol. Biol..

[63] Scranton M.A., Yee A., Park S.Y., Walling L.L. (2012). Plant leucine aminopeptidases moonlight as molecular chaperones to alleviate stress-induced damage. J. Biol. Chem..

[64] Nunes-Nesi A., Fernie A.R., Stitt M. (2010). Metabolic and Signaling Aspects Underpinning the Regulation of Plant Carbon Nitrogen Interactions. Mol. Plant.

[65] Zhang P.P., Ma G., Wang C.Y., Lu H.F., Li S.S., Xie Y.X., Ma D.Y., Zhu Y.J., Guo T.C. (2016). Effect of irrigation and nitrogen application on grain amino acid composition and protein quality in winter wheat. PLoS ONE.

[66] Zhen S.M., Deng X., Xu X.X., Liu N.N., Zhu D., Wang Z.M., Yan Y.M. (2020). Effect of high-nitrogen fertilizer on gliadin and glutenin subproteomes during kernel development in wheat (*Triticum aestivum* L.). Crop J..

[67] Schiene-Fischer C. (2015). Multidomain Peptidyl Prolyl cis/trans Isomerases. Biochim. Biophys. Acta.

[68] Dordas C. (2009). Dry matter, nitrogen and phosphorus accumulation, partitioning and remobilization as affected by N and P fertilization and source-sink relations. Eur. J. Agron..

[69] Yang J.C., Zhang J.H., Wang Z.Q., Xu G.M., Zhu Q.S. (2004). Activities of key enzymes in sucrose-to-starch conversion in wheat grains subjected to water deficit during grain filling. Plant Physiol..

[70] Yu Y.L., Zhu D., Ma C.Y., Cao H., Wang Y.P., Xu Y.H., Zhang W.Y., Yan Y. (2016). Transcriptome analysis reveals key differentially expressed genes involved in wheat grain development. Crop J..

[71] Zhang M., Lv D., Ge P., Bian Y., Chen G., Zhu G., Li X., Yan Y. (2014). Phosphoproteome analysis reveals new drought response and defense mechanisms of seedling leaves in bread wheat (*Triticum aestivum* L.). J. Proteom..

[72] Wang W., Scali M., Vignani R., Spadafora A., Sensi E., Mazzuca S., Cresti M. (2003). Protein extraction for two-dimensional electrophoresis from olive leaf, a plant tissue containing high levels of interfering compounds. Electrophoresis.

[73] Cao H., He M., Zhu C., Yuan L.L., Dong L.W., Bian Y.W., Zhang W.Y., Yan Y.M. (2016). Distinct metabolic changes between wheat embryo and endosperm during grain development revealed by 2D-DIGE-based integrative proteome analysis. Proteomics.

[74] Xu W.J., Lv H.J., Zhao M.M., Li Y.C., Qi Y.Y., Peng Z.Y. (2016). Proteomic comparison reveals the contribution of chloroplast to salt tolerance of a wheat introgression line. Sci. Rep..

[75] Shan Q.W., Wang Y.P., Li J., Gao C. (2014). Genome editing in rice and wheat using the CRISPR/Cas system. Nat. Protoc..

[76] Guo G., Lv D., Yan X., Subburaj S., Ge P., Li X., Hu Y., Yan Y. (2012). Proteome characterization of developing grains in bread wheat cultivars (*Triticum aestivum* L.). BMC Plant Biol..

[77] Livak K.J., Schmittgen T.D. (2011). Analysis of relative gene expression data using real-time quantitative PCR and the 2(−Delta Delta C(T)) method. Methods.

