# Supplementary figures and images for: Effects of Independent and Combined Water-Deficit and High-Nitrogen Treatments on Flag Leaf Proteomes during Wheat Grain Development
Source: Int J Mol Sci. 2020 Mar 19;21(6):2098. doi: 10.3390/ijms21062098 (PMC7139553; doi:10.3390/ijms21062098)

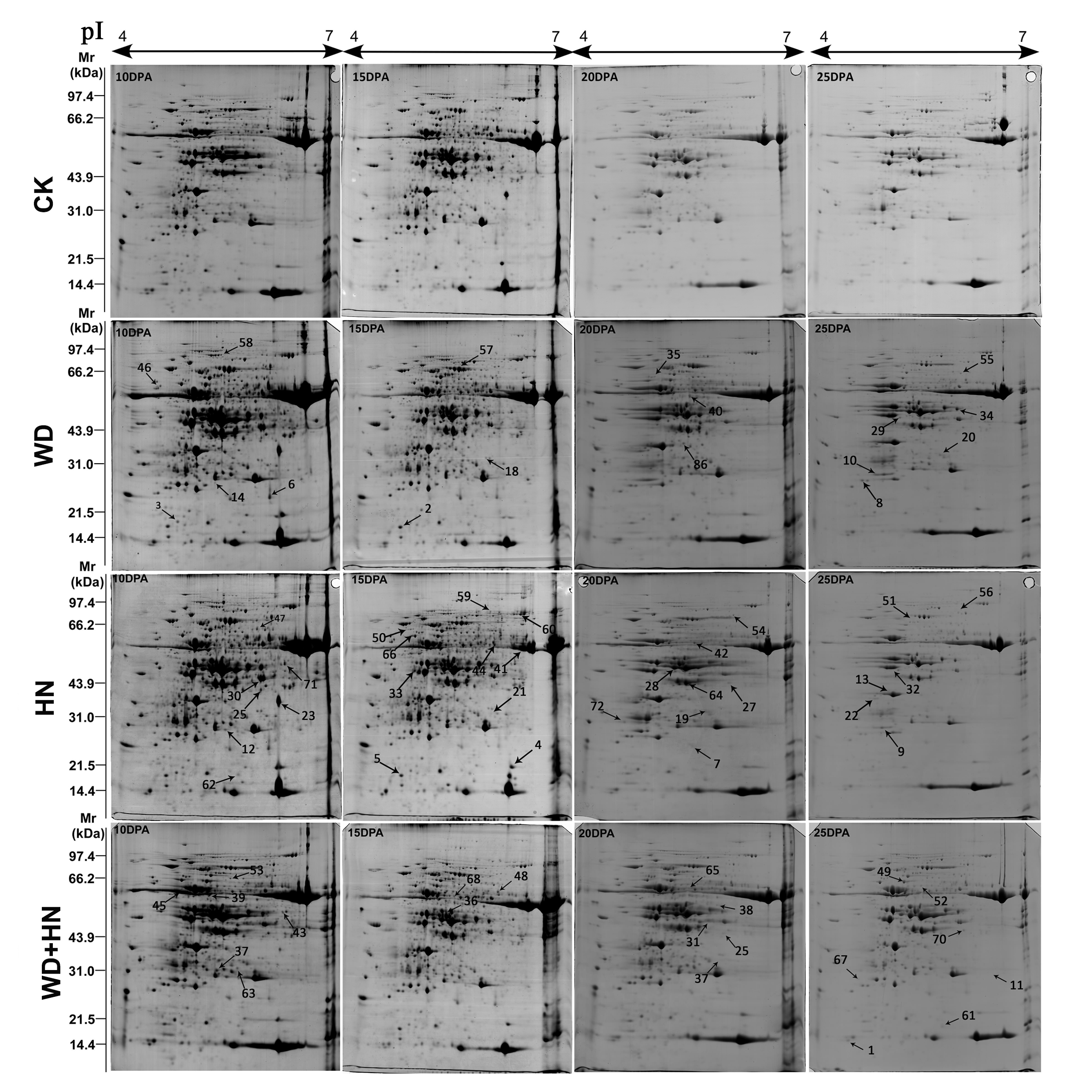

Supplement: Supplementary file 1 [file ijms-21-02098-s001.zip › Supplementary Materials/Figure S1.jpg]

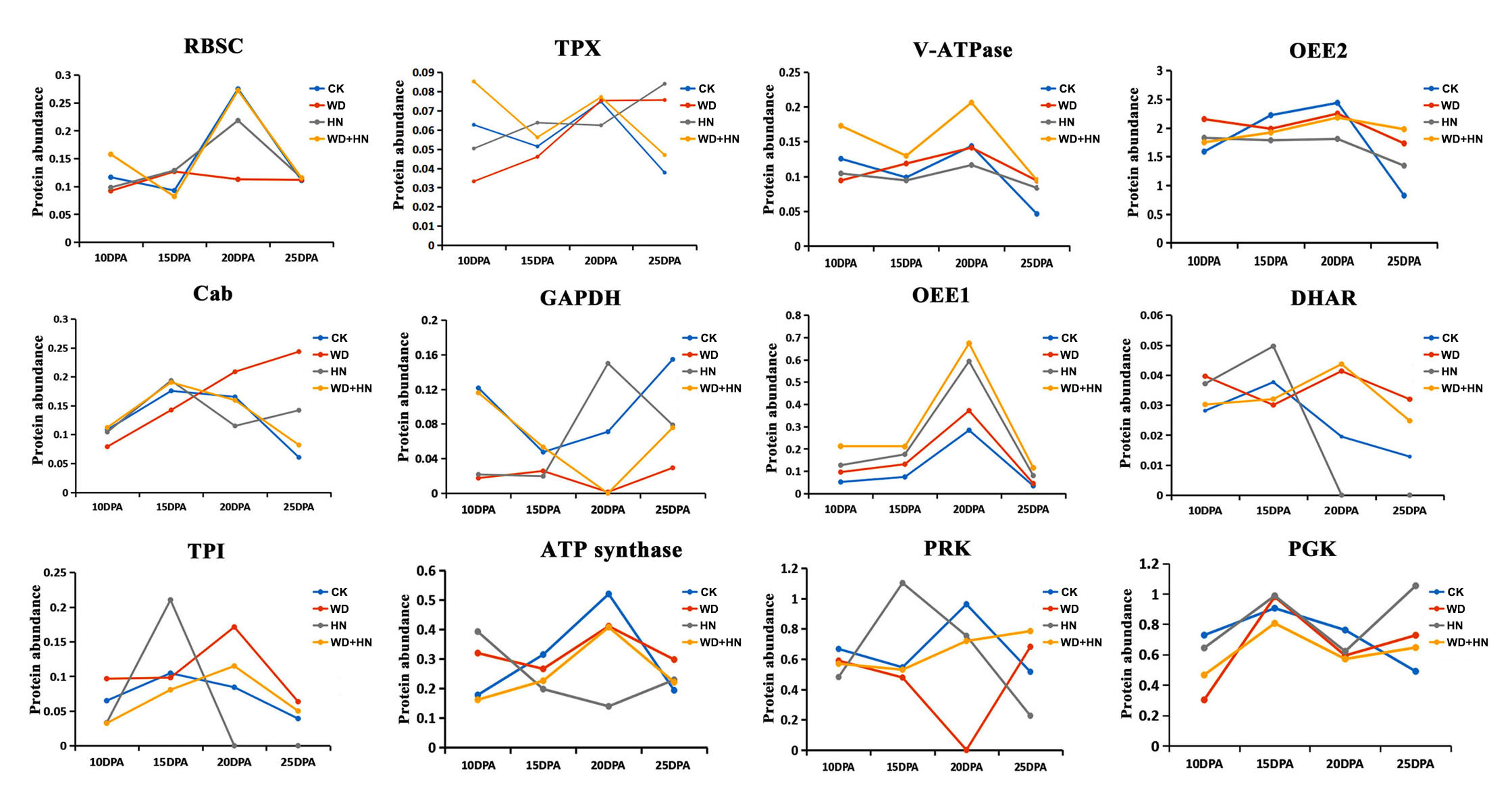

Supplement: Supplementary file 1 [file ijms-21-02098-s001.zip › Supplementary Materials/Figure S2.jpg]

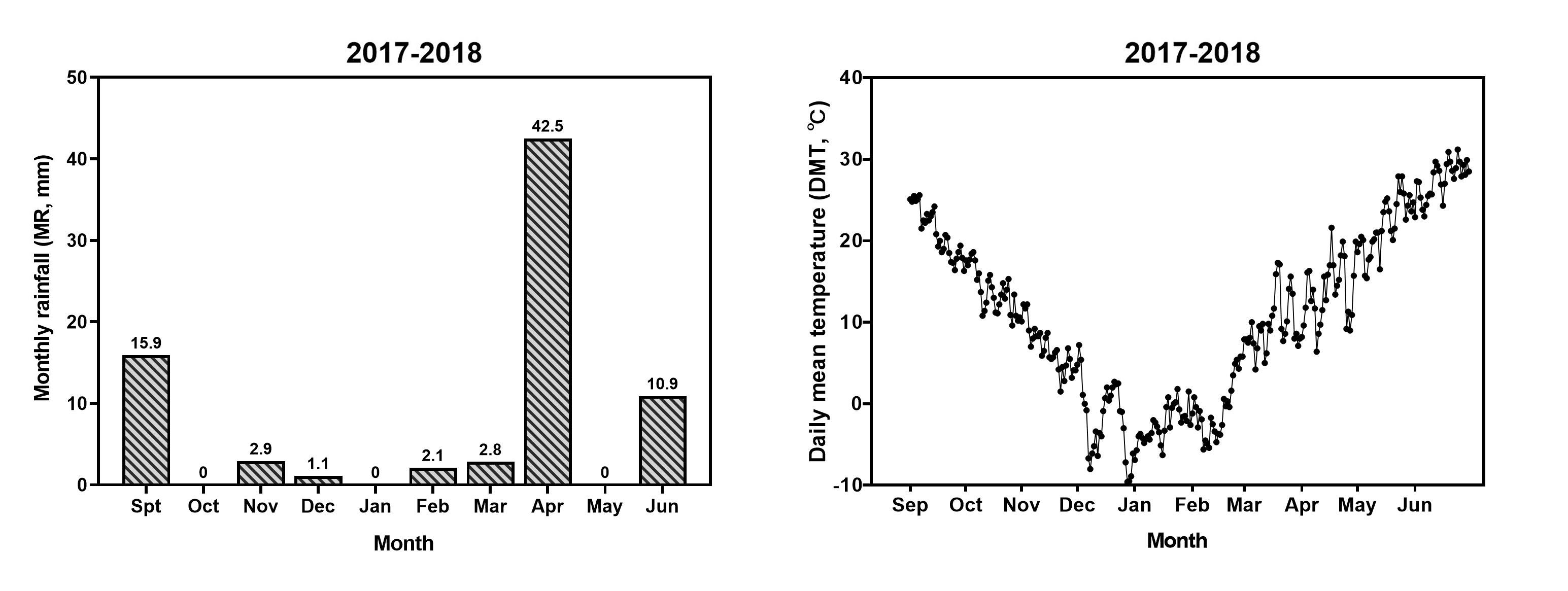

Supplement: Supplementary file 1 [file ijms-21-02098-s001.zip › Supplementary Materials/Figure S3.jpg]
